# Prognostic Value of Initial Assessment of Residual Hypoventilation Using Nocturnal Capnography in Mechanically Ventilated Neuromuscular Patients: A 5-Year Follow-up Study

**DOI:** 10.3389/fmed.2016.00040

**Published:** 2016-09-13

**Authors:** Adam Ogna, Julie Nardi, Helene Prigent, Maria-Antonia Quera Salva, Cendrine Chaffaut, Laure Lamothe, Sylvie Chevret, Djillali Annane, David Orlikowski, Frederic Lofaso

**Affiliations:** ^1^Service de Réanimation médicale et unité de ventilation à domicile, AP-HP, Hôpital Raymond Poincaré, Garches, France; ^2^Service de Physiologie-Explorations Fonctionnelles, AP-HP, Hôpital Raymond Poincaré, Garches, France; ^3^Unité du Sommeil, AP-HP, Hôpital Raymond Poincaré, Garches, France; ^4^Département de Biostatistique et Informatique Médicale, Hôpital Saint Louis, Paris, France; ^5^INSERM CIC 14.29, AP-HP, Hôpital Raymond Poincaré, Garches, France

**Keywords:** home mechanical ventilation, neuromuscular disease, restrictive respiratory failure, nocturnal hypoventilation, transcutaneous capno-oximetry, prognosis

## Abstract

**Background:**

Restrictive respiratory failure is a major cause of morbidity and mortality in neuromuscular diseases (NMD). Home mechanical ventilation (HMV) is used to treat hypoventilation, and its efficiency is mostly assessed by daytime blood gases or nocturnal oxygen saturation monitoring (SpO_2_). Non-invasive transcutaneous measure of CO_2_ (TcCO_2_) allows to directly assess nocturnal hypercapnia and to detect residual hypoventilation with a higher sensitivity than SpO_2_. We aimed to compare the prognostic value of nocturnal SpO_2_ and TcCO_2_ in ventilated adult NMD patients.

**Methods:**

All consecutive capno-oximetries performed between 2010 and 2011 in ventilated adult NMD patients were analyzed retrospectively. Concomitant blood gas analysis and lung function data were collected. Patients on oxygen therapy were excluded. Nocturnal hypoxemia and hypercapnia (using four different definitions) at baseline were compared in their ability to predict mortality and respiratory events requiring ICU admission during follow-up.

**Results:**

Data from 55 patients were analyzed (median age 28 [interquartile range: 25–36.5] years; 71% Duchenne muscular dystrophy; vital capacity 12 [7–27]% of predicted; 51% tracheostomy). Capno-oxymetry showed hypoxemia in 14.5% and hypercapnia in 12.7–41.8%, according to the used definition. Over a follow-up lasting up to 5 years (median 4.0 [3.6–4.5] years), we observed 12 deaths and 20 respiratory events requiring ICU admission. Hypercapnia was significantly associated with the study outcomes, with TcCO_2_ > 49 mmHg during ≥10% of the time being the best definition, while hypoxemia was not.

**Conclusion:**

Our data show for the first time that residual hypoventilation, assessed by capnometry, is significantly associated with negative outcomes in adult ventilated NMD patients, while oximetry is not. Accordingly, we suggest capnometry to be included in the systematic assessment of HMV efficiency in NMD patients.

**ClinicalTrials.gov Identifier:**

NCT02551406.

## Introduction

Neuromuscular diseases (NMD) are a heterogeneous group of rare diseases involving various components of the nervous system, including the respiratory muscles. The progressive weakness of the respiratory muscle pump results in restrictive respiratory failure and represents a major cause of morbidity and mortality in these pathologies (1–5). Long-term home mechanical ventilation (HMV) is effective to treat alveolar hypoventilation and currently represents one of the few available treatments improving the clinical course of NMD patients (2–4, 6–8). Once HMV is implemented, a regular follow-up is required to assure optimal tolerance and efficiency of the treatment. Besides the assessment of blood gases, HMV monitoring can be performed with different approaches with increasing complexity grade, ranging from simple tools, such as oximetry, to the most comprehensive sleep recording using in-hospital polysomnography (9). A management strategy was recently proposed, consisting of a simple initial screening based on nocturnal oxygen saturation monitoring (SpO_2_), followed by additional investigations in case of pathological findings (9). Non-invasive transcutaneous measure of CO_2_ (TcCO_2_) was found to have acceptable accuracy for estimating PaCO_2_ over several hours in stable home-ventilated patients (10, 11) and showed a higher sensitivity than SpO_2_ to detect residual hypoventilation in NMD patients (12, 13).

Current recommendations about monitoring and adjustment of HMV repose on expert opinions, since scientific literature comparing different strategies to assess HMV efficiency on their impact on hard clinical endpoints is still lacking (9, 14).

The aim of our study was to compare the prognostic value of SpO_2_ and TcCO_2_ in unselected ventilated adult NMD patients.

## Materials and Methods

### Patients

Data were collected retrospectively from the charts of NMD adults, followed at the Home Mechanical Ventilation Unit of the Raymond Poincare University Hospital, Garches, France. All consecutive capno-oximetries performed electively on mechanically ventilated patients between June 2009 and July 2011 were reviewed, and the oldest recording of each patient was retained for analysis. Patients on oxygen therapy were excluded. The study was conducted in accordance with the declaration of Helsinki and was approved by the French national regulatory board (CNIL, No. 1890638). ClinicalTrials.gov Identifier: NCT02551406.

### Capno-Oximetry

Overnight, continuous non-invasive TcCO_2_ and oxygen saturation (SpO_2_) were recorded simultaneously using a Digital Monitoring System (SenTec, Therwil, Switzerland) equipped with a combined Severinghaus-type TcCO_2_ electrode and SpO_2_ sensor (V-Sign, SenTec, Therwil, Switzerland). As recommended by the manufacturer, the electrode was calibrated in the built-in docking station before and after each measurement, using a service gas (mixture of 8% CO_2_, 12% O_2_, and 80% N_2_), allowing the measured TcCO_2_ values to be corrected for calibration drift. All studies were visually inspected to exclude periods with artifacts from the results, which represented in mean 1% of the recording time (range 0–4%).

### Daytime Blood Gases

According to routine clinical practice in the unit, daytime blood gas values were obtained on the morning following the capno-oxymetry. The blood sample was drawn at rest and immediately carried in an ice bag to the central chemical laboratory of the hospital, were it was analyzed using routine methods with stringent quality controls.

### Definition of Hypoventilation

Five different criteria were used to define residual hypoventilation during HMV, one reposing on oximetry and four on TcCO_2_ with different cut-offs:

– SpO_2_ < 90% during ≥10% of the total recording time (“hypoxemia”) (9, 12);
– peak TcCO_2_ > 49 mmHg (“hypercapnia[1]”) (7, 12);
– TcCO_2_ > 49 mmHg during ≥10% of the total recording time (“hypercapnia[2]”);
– peak TcCO_2_ > 55 mmHg (“hypercapnia[3]”);
– TcCO_2_ > 55 mmHg for ≥10 min or increase in TcCO_2_ ≥ 10 mmHg (in comparison to an awake supine value) to a value exceeding 50 mmHg for ≥10 min (“hypercapnia[4]”) (15).

### Outcomes

We considered mortality and acute respiratory events requiring ICU admission as outcomes of interest for the present analysis. Ventilated NMD patients are hospitalized at least annually for follow-up in our Unit, and at each visit, details on undercurrent hospitalizations in other hospitals are collected in the medical chart. In case of death, the furnisher of the home ventilator immediately reports the interruption of treatment to our Unit, and as much of informations as possible are collected.

### Statistical Analysis

Continuous variables were described by median and interquartile range (IQR); dichotomous or categorical variables were described by number of subjects and percentage. Single outcomes were analyzed by computing cumulative incidence curves and compared using Gray’s test, and the event-free survival was analyzed by computing Kaplan–Meier curves. Statistical analysis was conducted using R statistical software (R Core Team, www.r-project.org).

## Results

Data from 55 ventilated NMD adults were available. Most of the patients had Duchenne muscular dystrophy and a severe restrictive respiratory failure (median vital capacity 12% of predicted [IQR 7–27]), requiring 24-h mechanical ventilation in 45.5% of the cases. The characteristics of the study population are detailed in Table 1.

**Table 1:** **Characteristics of the study population**.

	*N* (%) or median [IQR]
**Parameters**	
Number of patients	55
Pathology (*N*, %)
– DMD	39 (70.9%)
– MD1	5 (9.1%)
– Other	11 (20.0%)
Age (years)	28 [25–36.5]
Weight (kg)	45.0 [36.0–60.5]
BMI (kg/m^2^)	17.0 [14.6–24.2]
Follow-up (years)	4.0 [3.6–4.5]
Deaths (*N*, %)	12 (21.8%)
ICU admissions (*N*, %)	20 (36.4%)
**Respiratory parameters**	
VC sitting (%pred)	12 [7–27]
VC supine (%pred)	10 [5–20]
PI max (cmH_2_O)	12 [3–27]
PE max (cmH_2_O)	10 [5–24]
**Mechanical ventilation**	
Volumetric mode (*N*, %)	40 (72.7%)
Tracheostomy (*N*, %)	28 (50.9%)
Daily HMV duration (h)	22.5 [9.0–24.0]
**Daytime blood gases**	
pH	7.42 [7.39–7.47]
PaO_2_ (kPa)	11.3 [9.3–15.0]
PaCO_2_ (kPa)	4.78 [4.04–5.46]
Bicarbonates (mmol/l)	24.7 [21.6–27.2]
**Nocturnal Capno-Oximetry**	
Duration of the recording (min)	473 [459–475]
Mean oxygen saturation (%)	96 [95–98]
Mean nocturnal TcCO_2_ (mmHg)	39.8 [30.4–45.0]
Max nocturnal TcCO_2_ (mmHg)	48.1 [37.4–52.5]

*DMD, Duchenne muscular dystrophy; MD1, myotonic dystrophy type 1 (Steinert’s disease); BMI, body mass index; VC, vital capacity; %pred, percentage of the predicted value; PI max, maximal inspiratory pressure; PE max, maximal expiratory pressure; HMV, home mechanical ventilation; PaCO_2_, partial arterial pressure of CO_2_; PaO_2_, partial arterial pressure of O_2_; TcCO_2_, transcutaneous measure of CO_2_; IQR, interquartile range*.

### Capno-Oximetry

**Figure 1:**
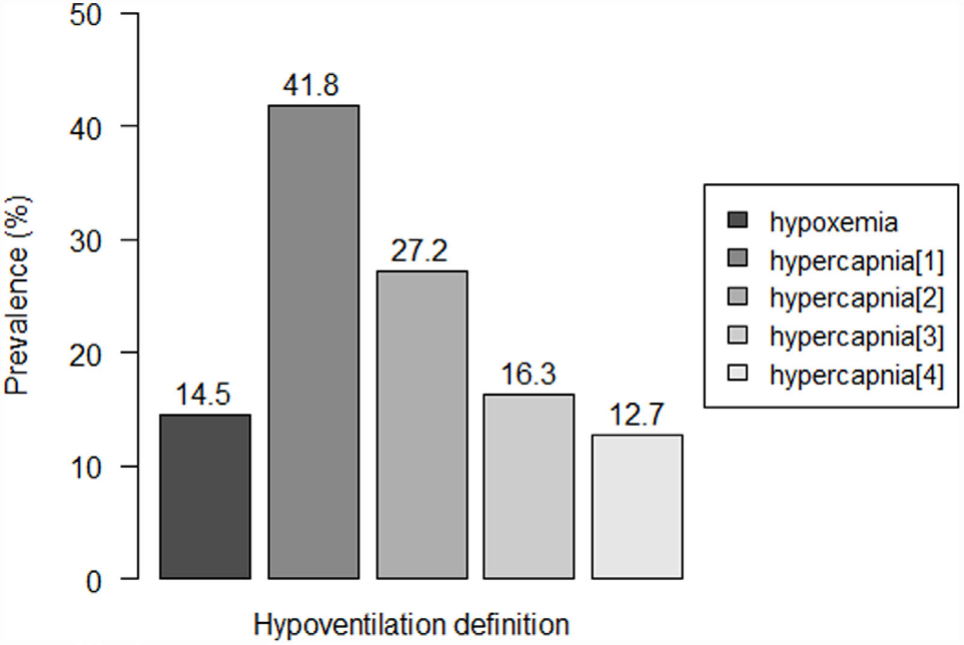
**Prevalence of residual hypoventilation according to the different definitions**. Hypoventilation definitions: “hypoxemia”: oxygen saturation (SpO_2_) < 90% during ≥10% of the total recording time; “hypercapnia[1]”: peak transcutaneous CO_2_ (TcCO_2_) > 49 mmHg; “hypercapnia[2]”: TcCO_2_ > 49 mmHg during ≥10% of the total recording time; “hypercapnia[3]”: peak TcCO_2_ > 55 mmHg, “hypercapnia[3]”: TcCO_2_ > 55 mmHg for ≥10 min, or increase in TcCO_2_ ≥ 10 mmHg in comparison to baseline to a value exceeding 50 mmHg for ≥10 min.

Eight patients (14.5%) were detected to have hypoxemia, while prevalence of hypercapnia ranged from 12.7 to 41.8%, according to the used criterion (Figure 1). Three to seven patients showed both hypoxemia and hypercapnia, depending on the criterion used to define hypercapnia.

### Outcomes

Over a follow-up period lasting up to 5 years (median 4.0 [IQR 3.6–4.5] years), we observed 12 deaths and 20 acute respiratory events requiring ICU admission. The overall mortality was 5.5% at 2 years and 33.0% at 5 years, without significant difference between the study groups.

**Figure 2:**
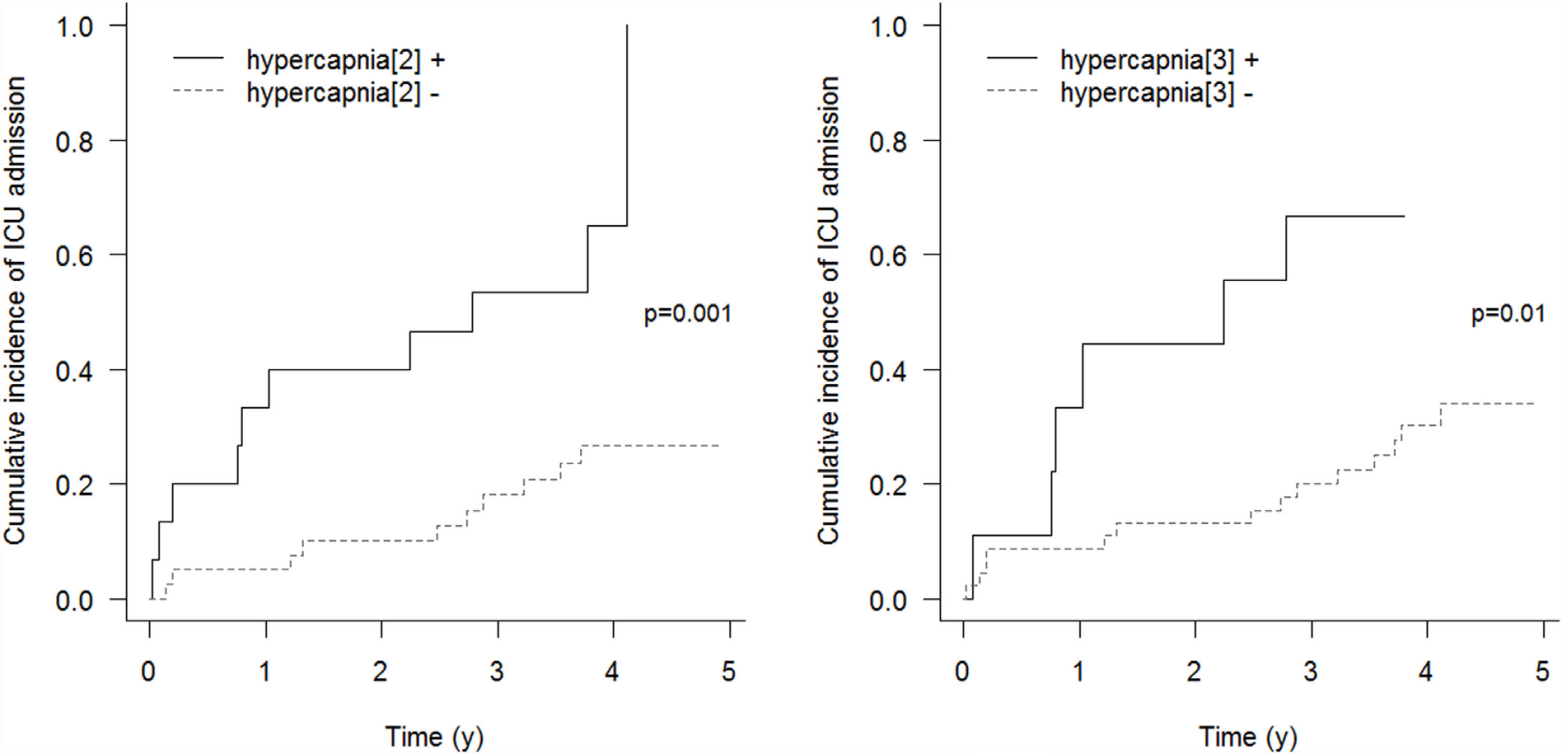
**Cumulative incidence of respiratory events requiring ICU admission**. “hypercapnia[2]”: TcCO_2_ > 49 mmHg during ≥10% of the total recording time; “hypercapnia[3]”: peak TcCO_2_ > 55 mmHg.

**Figure 3:**
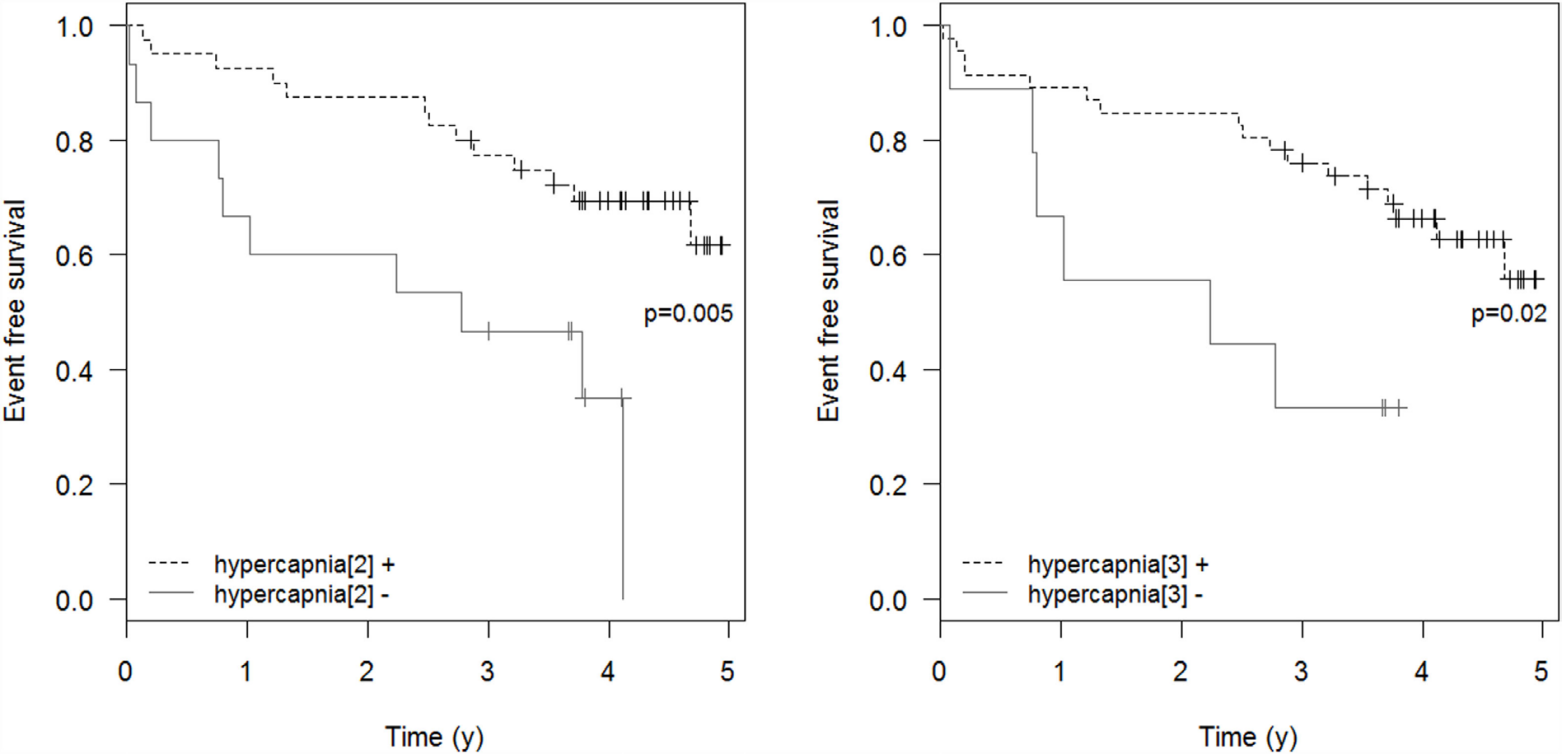
**Event-free survival**. Event-free survival: time to the composite endpoint ICU admission or death. “hypercapnia[2]”: TcCO_2_ > 49 mmHg during ≥10% of the total recording time; “hypercapnia[3]”: peak TcCO_2_ > 55 mmHg.

Respiratory events requiring ICU admission were significantly associated with residual hypercapnia (Figure 2), defined both as TcCO_2_ > 49 mmHg during ≥10% of the total recording time (“hypercapnia[2],” *p* = 0.001) and peak TcCO_2_ > 55 mmHg (“hypercapnia[3],” *p* = 0.01), while peak TcCO_2_ > 49 mmHg (“hypercapnia[1]”) showed a borderline significant association (*p* = 0.06). Neither the definition of hypercapnia suggested by the American Academy for Sleep Medicine (AASM) (“hypercapnia[4]”: TcCO_2_ > 55 mmHg for ≥10 min or increase in TcCO_2_ ≥ 10 mmHg in comparison to an awake supine value to a value exceeding 50 mmHg for ≥10 min) nor hypoxemia were associated with respiratory events (*p* = 0.15 and *p* = 0.11, respectively). Similar results were found exploring the event (mortality or ICU admission)-free survival of the patients (*p* = 0.005 for “hypercapnia[2]” definition and *p* = 0.02 for the “hypercapnia[3]” definition, Figure 3).

However, using “hypercapnia[2]” as a diagnostic criterion identified almost twofold inefficiently ventilated patients as compared with the “hypercapnia[3]” criterion.

## Discussion

We report the first data on the prognostic value of different tools to identify residual hypoventilation in mechanically ventilated neuromuscular patients. Analyzing an unselected NMD population, we found that inefficiently ventilated patients are at increased risk of mortality or ICU admission when compared with efficiently ventilated patients. Comparing two monitoring techniques, we observed marked differences in the detection rate of residual hypoventilation and showed that hypercapnia was significantly associated with the risk of respiratory events requiring ICU admission in this population, while hypoxemia was not. Our results have practical consequences for the daily clinical practice, since the evaluation of HMV efficiency belongs to the crucial tasks in the management of mechanically ventilated patients.

Restrictive respiratory failure represents one of the leading causes of morbidity and mortality in NMD (1–5) and manifests as hypoventilation, which can be effectively treated by HMV (7, 16–18). The evidence supporting the use of HMV in NMD, although mostly based on observational studies, is consistent suggesting that the treatment for hypoventilation with HMV improves clinical symptoms and quality of life and reduces the risk of unplanned hospitalization and mortality, compared with no ventilation (6–8, 17, 19, 20).

In the absence of scientific evidence supporting the choice of the best strategy to assess HMV efficiency, current recommendations on the adjustment of HMV repose on expert opinions, indicating the correction of hypoventilation as the main objective. Since the definition of hypoventilation is neither univocal nor based on prognostic studies (1, 15, 21, 22), an additional assumption is made by consensus, making the recommendations even more delicate. As such, the European SomnoNIV Group suggests an algorithm for monitoring HMV, which includes oximetry as the first screening step to identify patients who require further nocturnal investigations, and suggests a mean nocturnal SpO_2_ over 90% for at least 90% of the total recording time as a goal (9). For its part, the AASM recommend in its 2010 best clinical practice guidelines to adapt the ventilator support if hypoventilation is present for ≥10 min (14).

Our results support the choice of correcting hypoventilation as a therapeutic goal, given that hypercapnia – and thus residual hypoventilation – seems to be significantly associated with the risk of respiratory events in the following few years. In the meantime, our data underline once more the lack of sensitivity of SpO_2_ to identify nocturnal hypoventilation in NMD patients. In contrast to chronic obstructive pulmonary disease (COPD), NMD patients mostly suffer from exclusive restrictive respiratory failure without ventilation–perfusion mismatch (23). As a consequence, NMD patients have higher baseline PaO_2_ values than COPD patients, being on the flat portion of the oxyhemoglobin dissociation curve, so that the small decrease in PaO_2_ occurring during alveolar hypoventilation is poorly reflected by changes in the SpO_2_ (24, 25).

Recent evidence showed that TcCO_2_ can be an accurate proxy for PaCO_2_ in long-term mechanically ventilated patients, with the advantage to detect episodes of transient hypoventilation, that are not detected by punctual blood gazes (10, 11). Our findings add to this evidence, suggesting that TcCO_2_ monitoring may also have a prognostic interest in stable, home-ventilated NMD patients. The use of TcCO_2_ opens the possibility to assess the ventilation’s efficacy directly and repeated at home, allowing a simplification in the management of HMV. However, despite the recent technical improvements of capnometry devices, TcCO_2_ accuracy is strongly dependent on appropriate handling and knowledge of the equipment and procedure (10).

The main limitation of our study is linked to its retrospective design, which does not allow to conclude on a causative role of residual hypoventilation in the observed increased morbidity. Although inefficiently ventilated patients may reasonably have an increased respiratory frailty and, thus, be prone to develop severe respiratory failure even after a minor infectious event, it is in effect possible that residual hypoventilation represents a marker of a more severe disease stage rather than an independent risk factor for future morbidity, even if we could observe no difference in the severity of disease between the study groups (Table 2). Furthermore, only a prospective trial would allow inferring on the prognostic effect of ventilation modifications to correct residual hypoventilation. The second point is the lack of an objective sleep assessment during capno-oximetry, so that prolonged periods of wake during the recording period could increase the rate of false negatives. A further limitation, which is linked to the fact that NMD are rare disorders, is the relatively small sample size, with analyses being performed on few patients.

**Table 2:** **Disease severity according to the study subgroup**.

	hypercapnia[2]+	hypercapnia[2]−	hypercapnia[3]+	hypercapnia[3]−
**Parameters**				
Number of patients	15	40	9	46
Age (years)	27 [23.5–31]	29.5 [25–37]	29 [26–30]	28 [25–37]
Weight (kg)	37.0 [28.8–53.0]	48.0 [38.8–60.8]	37.0 [28.5–61.0]	46.5 [38.0–60.0]
BMI (kg/m^2^)	14.5 [10.4–17.6]^a^	18.7 [15.5–24.6]	12.3 [10.3–18.4]	17.5 [15.4–24.4]
**Respiratory parameters**				
VC sitting (%pred)	10 [7.5–29]	13 [7–25.5]	10 [8.5–36]	12.5 [7–24]
VC supine (%pred)	6 [5–9]	6 [5–21]	6 [5–8]	11 [5–21.5]
PI max (cmH_2_O)	10 [7–23.5]	14 [2–27]	10.5 [8–21]	12 [2.5–27]
PE max (cmH_2_O)	10 [7.5–24]	10 [3–24]	10.5 [9–21]	10 [3.5–24]
**Mechanical ventilation**				
Volumetric mode (*N*, %)	10 (66.7%)	30 (75.0%)	7 (77.8%)	33 (71.7%)
Tracheostomy (*N*, %)	7 (46.7%)	21 (52.5%)	5 (55.6%)	23 (50.0%)
Daily HMV duration (h)	21.0 [9.0–24.0]	24.0 [11.2–24.0]	23.0 [9.0–24.0]	24.0 [9.0–24.0]

*Values are expressed as *N* (%) of median [interquartile range]*.

*^a^p < 0.05*.

*BMI, body mass index; VC, vital capacity; %pred, percentage of the predicted value; PI max, maximal inspiratory pressure; PE max, maximal expiratory pressure; HMV, home mechanical ventilation*.

*“hypercapnia[2]”: TcCO_2_ > 49 mmHg during ≥10% of the total recording time; “hypercapnia[3]”: peak TcCO_2_ > 55 mmHg*.

Another point needs to be addressed in the absence of a consensus about the best nocturnal TcCO_2_ criterion for assessing HMV efficiency, we arbitrarily defined the cut-offs used in our study, to ease comparability with previous published data (7, 12). The choice of a different definition such as a mean TcCO_2_ above 50 mmHg would, for example, have led to the identification of only four patients in the “hypoventilation” group, thus having for consequence the overlook of several patients who showed an increased morbidity risk according to the presented results. Interestingly, the hypercapnia definition suggested in the AASM 2012 guidelines (15) resulted not discriminatory for an increased risk in our population.

## Conclusion

Our data show for the first time that residual hypoventilation, assessed by capnometry, is significantly associated with negative outcomes in adult ventilated NMD patients, while simple oximetry is not. These findings may have practical consequences on the strategies used to assess HMV efficiency in NMD patients and provide support for an appropriately designed trial using capnography to investigate its predictive capacity for negative outcomes in NMD.

## Author Notes

*Notation of prior abstract publication/presentation*: An abstract with preliminary data has been presented at the European Respiratory Society (ERS) congress, Amsterdam (Netherlands), 26–30 September 2015.

## Author Contributions

AO, JN, HP, M-AS, DA, DO, and FL: designed the experiment; AO, JN, HP, M-AS, LL, DA, and DO: conducted the research; AO, CC, SC, DO, and FL: analyzed the data and performed the statistical analyses; AO, HP, LL, and DO: wrote the manuscript. All authors had full access to all of the data (including statistical reports and tables) in the study, revised the manuscript for important intellectual content, and approved the final version of the manuscript.

## Conflict of Interest Statement

All the authors declare that they have no conflict of interest related to the present work to disclose. The Service de Physiologie-Explorations Fonctionnelles of Garches received research funds from ResMed France, not related to the present work. The CIC 14.29 of Garches received research funds from BREAS Medical for a project on end-tidal CO_2_.

## Abbreviations

CO_2_, carbon dioxide; DMD, Duchenne muscular dystrophy; HMV, home mechanical ventilation; MD1, myotonic dystrophy type 1 (Steinert’s disease); NMD, neuromuscular diseases; PaCO_2_, partial arterial pressure of CO_2_; PE max, maximal expiratory pressure; PI max, maximal inspiratory pressure; SpO_2_, oxygen saturation; TcCO_2_, transcutaneous measure of CO_2_; VC, respiratory vital capacity.
